# Posttreatment with PaPE-1 Protects from Aβ-Induced Neurodegeneration Through Inhibiting the Expression of Alzheimer’s Disease-Related Genes and Apoptosis Process That Involves Enhanced DNA Methylation of Specific Genes

**DOI:** 10.1007/s12035-023-03819-5

**Published:** 2023-12-08

**Authors:** Bernadeta A. Pietrzak-Wawrzyńska, Agnieszka Wnuk, Karolina Przepiórska-Drońska, Andrzej Łach, Małgorzata Kajta

**Affiliations:** Laboratory of Neuropharmacology and Epigenetics, Department of Pharmacology, Maj Institute of Pharmacology, Polish Academy of Sciences, Smetna Street 12, 31–343 Krakow, Poland

**Keywords:** Neuroprotection, Primary neocortical cell cultures, Non-nuclear estrogen receptor signaling, Caspases, Amyloid-β, Alzheimer’s disease

## Abstract

Targeting the non-nuclear estrogen receptor (ER) signaling has been postulated as novel therapeutic strategy for central nervous system pathologies. Recently, we showed that newly designed PaPE-1 (Pathway Preferential Estrogen-1), which selectively activates ER non-nuclear signaling pathways, elicited neuroprotection in a cellular model of Alzheimer’s disease (AD) when it was applied at the same time as amyloid-β (Aβ). Since delayed treatment reflects clinical settings better than cotreatment does, current basic study proposes a novel therapeutic approach for AD that relies on a posttreatment with PaPE-1. In this study, mouse neuronal cell cultures treated with preaggregated Aβ_1-42_ (10 µM) showed the presence of extracellular Aβ_1-42_, confirming the adequacy of the AD model used. We are the first to demonstrate that a 24-h delayed posttreatment with PaPE-1 decreased the degree of Aβ-induced neurodegeneration, restored neurite outgrowth, and inhibited the expression of AD-related genes, i.e., *Rbfox*, *Apoe*, *Bace2*, *App*, and *Ngrn*, except for *Chat*, which was stimulated. In addition, PaPE-1 elicited anti-apoptotic effects by inhibiting Aβ-induced caspase activities as well as attenuating apoptotic chromatin condensation, and in these ways, PaPE-1 prevented neuronal cell death. Posttreatment with PaPE-1 also downregulated the Aβ-affected mRNA expression of apoptosis-specific factors, such as *Bax*, *Gsk3b*, *Fas*, and *Fasl*, except for *Bcl2*, which was upregulated by PaPE-1. In parallel, PaPE-1 decreased the protein levels of BAX, FAS, and FASL, which were elevated in response to Aβ. PaPE-1 elicited a decrease in the BAX/BCL2 ratio that corresponds to increased methylation of the *Bax* gene. However, the PaPE-1-evoked *Bcl2* gene hypermethylation suggests other PaPE-1-dependent mechanisms to control Aβ-induced apoptosis.

**Supplementary Information:**

The online version contains supplementary material available at 10.1007/s12035-023-03819-5.

## Introduction

According to the World Health Organization, more than 55 million people suffer from dementia worldwide, and this number is predicted to increase by 10 million every year. Alzheimer’s disease (AD) is the most common cause of dementia, accounting for 60–70% of all its cases. AD is an age-related neurodegenerative disorder with characteristic progressive deterioration of memory and cognitive functions. Since it affects not only patients but also their relatives, i.e., unpaid caregivers, it is a serious economic problem in modern society. Molecular changes initiate AD long before clinical manifestation. These changes include extracellular amyloid-β (Aβ) plaques and intracellular neurofibrillary tangles along with synaptic and neural loss. Aβ peptides are derived from amyloid precursor protein (APP) following cleavage by β- and γ-secretases [1]. Then, the monomeric Aβ is secreted into the extracellular space, where it forms toxic oligomers and fibrils. The main mechanisms of Aβ toxicity involve cell membrane destruction, mitochondrial damage, Ca^2+^ homeostasis dysregulation, alteration of receptor signaling, and aberrant activation of certain apoptotic factors [2, 3].

The loss of neurons is inversely related to patients’ cognitive skills. Extensive neuronal loss in AD is attributed to apoptosis [4]. Apoptosis is a form of programmed cell death that eventually leads to the activation of apoptosis-specific cysteine-aspartic proteases, i.e., caspases. These enzymes can be divided into two categories: inducing (caspase-2, caspase-8, caspase-9, caspase-10, and caspase-12) and executive (caspase-3, caspase-6, and caspase-7) [5]. Extracellular and intracellular pathways can initiate apoptosis. The intracellular apoptotic pathway involves, among others, BCL2 and BAX, associated with the formation of pores in the mitochondrial membrane that cause cytochrome c release and caspase-9 activation [6]. The extracellular apoptotic pathway starts with the activation of death receptors by cognate ligands (e.g., FAS and FASL) and then leads to caspase-8 activation [7]. Inducing caspases (caspase-8 and caspase-9) activate executive caspase-3, which leads to cell death. Caspases are known to be engaged in AD pathology [8]. In addition to its role in apoptotic cell death, caspase-3 is also able to cleave both APP and tau [9–12] and induce tau hyperphosphorylation [13]. In the nervous system, caspases are also vital for a plethora of physiological functions, including but not limited to neural tube shaping, dendritic pruning, axon guidance, synaptogenesis, and maintenance of synaptic plasticity [14]. Caspase-8, caspase-9, and caspase-3 were also shown to be engaged in the neurite outgrowth process [15].

There is an urgent need to find novel pharmaceuticals that effectively target AD. Currently available pharmacological approaches are based on cholinesterase inhibition (donepezil, rivastigmine, and galantamine) and NMDA receptor antagonism (memantine) [16]. Although these drugs may help control some cognitive and behavioral symptoms, they do not alter disease progression. In addition, the Food and Drug Administration (FDA) has recently approved two new Aβ-targeting monoclonal antibodies, aducanumab and lecanemab, but their usefulness in clinical settings remains to be determined. Despite the fact that these pharmaceuticals may delay clinical progression of AD, their usage can also cause undesirable effects such as microhemorrhages and brain edema. None of the abovementioned drugs target apoptosis-related neuronal loss. Since direct caspase inhibitors are not able to distinguish between physiological and pathological caspase activation, more selective approaches are required [17]. Activation of estrogen receptors (ERs) in the nervous system has been repeatedly demonstrated to be anti-apoptotic as well as to positively impact neural plasticity and synapse formation [7]. ER signaling can be divided into nuclear and non-nuclear. In the nuclear signaling, ESR1/ERα and ESR2/ERβ act as ligand-activated transcription factors. The non-nuclear signaling is often associated with activation of membrane-associated ERs that can be divided into G protein-coupled receptors (GPCRs), which include GPER1, Gq-mER, and ER-X, and non-GPCR mERα and mERβ. Many studies indicate that activation of nuclear ER signaling can lead to cardiovascular problems and hormonal-dependent cancers; for this reason, targeting non-nuclear ER signaling pathways, mainly via mERα and mERβ, appears to be a much safer but still effective alternative [18]. Pathway preferential estrogen-1 (PaPE-1; (S)-5-(4-hydroxy-3,5-dimethyl-phenyl)-indan-1-ol) has been designed to preferentially activate ER non-genomic signaling without stimulating direct nuclear signaling [19].

Compared to estradiol, PaPE-1 has greatly lowered binding affinities to ERα and ERβ and greatly increased dissociation rate from ERα [19]. Activation of the ERα by PaPE-1 lasts less than 1 min which is enough to initiate signaling cascades, but too short to evoke direct genomic effects [19]. PaPE-1 does not cause the recruitment of ERα or ERK2 to chromatin but stimulates the recruitment of RNA Pol II [19]. Moreover, in contrast to estradiol, ERα complexes with PaPE-1 do not bind SRC3 (steroid receptor coactivator 3) [19]. What is of importance, PaPE-1 activity is lost in ERα-knockout mice [19]. PaPE-1 selectively activates extranuclear-initiated ER-regulated genes, which was shown by *LRRC54* stimulation, but does not activate the nuclear-initiated ER gene target *PgR* [19]. In the MCF-7 cell line, PaPE-1 has been shown to selectively activate MAPK and mTOR signaling [19].

PaPE-1 has already been demonstrated to provide beneficial metabolic and vascular effects without stimulating reproductive tissues [19]. In addition, PaPE-1 administered before middle cerebral artery occlusion (MCAO) has been shown to attenuate stroke severity and neuroinflammation and promote functional recovery [20]. PaPE-1 has also been shown to be an effective neuroprotective agent in the posttreatment paradigm in hypoxic and ischemic models in vitro [21]. Our previous study demonstrated that cotreatment with PaPE-1 is a promising approach against AD, as shown in an in vitro model based on Aβ-induced neurotoxicity [22].

Since a posttreatment paradigm is the most relevant approach in AD treatment, the present study aims to identify the neuroprotective potential and mechanisms of action of PaPE-1 as a posttreatment therapy in an Aβ-based in vitro model of AD.

## Materials and Methods

### Materials

Phosphate-buffered saline (PBS) was obtained from Biomed Lublin (Lublin, Poland). Neurobasal medium and B27 were purchased from Gibco (Grand Island, NY, USA). Culture plates for cell cultures were obtained from Techno Plastic Products AG (Trasadingen, Switzerland), Corning (Corning, NY, USA) and Ibidi (Gräfelfing, Germany). Fetal bovine serum (FBS), *L*-glutamine, dimethyl sulfoxide (DMSO), 4-(2-hydroxyethyl)-1-piperazineethanesulfonic acid (HEPES), 3-[(3-cholamidopropyl)-dimethylammonio]-1-propanesulfonate hydrate (CHAPS), ammonium persulfate, N,N,N′,N′-tetramethylethane-1,2-diamine (TEMED), 2-amino-2-(hydroxymethyl)-1,3-propanediol (Trizma base), sodium deoxycholate, DL-dithiothreitol, poly-*L-*ornithine, (S)-5-(4-hydroxy-3,5-dimethyl-phenyl)-indian-1-ol (PaPE-1), Tween 20, radioimmunoprecipitation assay buffer (RIPA) and protease inhibitor cocktail, SP600125, and GenElute™ Mammalian Genomic DNA Miniprep Kits were purchased from Sigma‒Aldrich (St. Louis, MO, USA). Amyloid-β was obtained from rPeptide (Watkinsville, GA, USA). The RNeasy Mini Kit, EpiTect MethyLight PCR Kit, and EpiTect Bisulfite Kit were obtained from Qiagen (Hilden, Germany). High-Capacity cDNA-Reverse Transcription Kit, Neurite Outgrowth Staining Kit, TaqMan Gene Expression Master Mix, and TaqMan probes for specific genes encoding *Hprt*, *Actb*, *Gapdh*, *Fas*, *Fasl*, *Bax*, *Bcl2*, *Gsk3b*, *Rbfox*, *Ache*, *Apoe*, *Chat*, *Bace1*, *Bace2*, *Mapt*, *App*, *Rcan1*, *Ide*, and *Ngrn* were obtained from Thermo Fisher Scientific (Waltham, MA, USA). Sodium dodecyl sulfate (SDS), Bradford reagent, Laemmli sample buffer, and 0.5 M Tris–HCL buffer 4–15% Mini-PROTEAN TGX Precast Protein Gels were purchased from Bio-Rad Laboratories (Munich, Germany). The ROS-Glo™ H_2_O_2_ assay was obtained from Promega (Madison, WI, USA). JC-10 Mitochondrial Membrane Potential Assay Kit, Z-IETD-FMK, Z-LEHD-FMK, TDZD 8, and SB203580 were purchased from Abcam (Cambridge, UK). 2-Mercaptoethanol was obtained from Carl Roth GmbH + Co. KG (Karlsruhe, Germany). Immobilon-P membranes were purchased from Millipore (Bedford, MA, USA). Fluoro-Jade C was obtained from Biosensis Pty Ltd. (Thebarton, Australia). Antibodies used for western blot and immunofluorescence staining were as follows: anti-GAPDH (MAB374), anti-BCL-2 (SAB5700155), and anti-MAP2 (M9942)-obtained from Sigma‒Aldrich (St. Louis, MO, USA); anti-amyloid β (bs-0107R)-purchased from Thermo Fisher Scientific (Waltham, MA, USA); and anti-BAX (SC-7480), anti-GSK3β (sc-9166), anti-FAS (sc-74540), anti-FASL (sc-19681), and anti-MAP2 (sc-20172)-purchased from Santa Cruz Biotechnology Inc. (Santa Cruz, CA, USA).

### Primary Neuronal Cell Culture

Primary neuronal cell cultures were established using Swiss CD1 mice obtained from Charles River Laboratory (Germany) as described previously [23, 24]. Cortices acquired from embryos were fragmented and incubated with 0.1% trypsin at 37 °C for 15 min. Next, the cells were centrifuged for 5 min at 1500 × *g* in medium with 10% FBS. The obtained neuronal cells were then seeded on multiwell plates coated with poly-*L*-ornithine (0.1 mg/ml) at a density of ~2.0 × 10^5^ and further cultured in Neurobasal medium with the addition of *L*-glutamine, B27, and an antibiotic cocktail containing penicillin and streptomycin. Cells were cultured for 7 days at 37 °C with humidified air with a CO_2_ concentration of 5%. For the first 2 days, the cells were cultured with FBS added to the culture medium. All animals used in the research were maintained according to the principles of the Three Rs in compliance with European Union Legislation (Directive 2010/63/EU, amended by Regulation (EU) 2019/1010).

### Treatment

Preparation of Aβ_1-42_ was conducted as previously described [22]. Non-specific aggregation of Aβ_1-42_ was negated using HFIP (hexafluoroisopropanol). Next, HFIP was removed under N_2_ flux, and Aβ_1-42_ was dissolved using DMSO to prepare a stock solution, which was further dissolved in culture medium. The obtained solution of Aβ_1-42_ was then incubated overnight to induce specific aggregation and then used to treat the cell cultures. After 24-h treatment with preaggregated Aβ_1-42_ (10 µM), PaPE-1 (at concentrations of 5 and 10 µM) was applied for the following 6 h. To determine the contribution of apoptotic signaling, we applied Z-IETD-FMK (caspase-8 inhibitor; 40 µM), Z-LEHD-FMK (caspase-9 inhibitor; 40 µM), TDZD 8 (GSK3β inhibitor; 1 µM), SP600125 (JNK inhibitor; 1 µM), and SB 203580 (p38 MAPK inhibitor; 1 µM) to cells treated with Aβ. DMSO was used as a solvent for all compounds at concentrations not exceeding 0.1% in the culture medium.

### Assessment of Caspase Activity

In this research, we assessed the activity of caspase-3, caspase-8, and caspase-9. The assessment procedure is identical for each of the enzymes, with the only difference being the substrate used and, consequently, the product of the reaction.

For caspase-3, the colorimetric substrate was Ac-DEVD-*p*NA (N-acetyl-asp-glu-val-asp-*p*-nitroanilide, Sigma‒Aldrich, St. Louis, MO, USA), and the product was *p*-nitroanilide. For caspase-8, the substrate was Ac-VETD-AMC (Ac-val-glu-thr-asp-AMC), and the product was 7-amino-4-methylcoumarin. For caspase-9, the substrate was Ac-DL-Leu-DL-Glu-DL-His-DL-Asp-*p*NA, and the product is *p*-nitroanilide.

The activity of caspases was measured as described earlier [25]. Samples were first incubated with CAB (*Caspase Assay Buffer*) for 15 min at 4 °C and then with substrate specific for each caspase for 60 min at 37 °C. The levels of caspase reaction products were measured with an Infinite M200PRO microplate reader (Tecan, Switzerland) at excitation = 400 nm and emission = 530 nm for caspase-8 activity measurement and absorbance = 405 nm for caspase-3 and caspase-9 activity measurements. The obtained data were analyzed with i-control software and normalized to the blank, and the final results are presented as a percentage of the control ± SEM.

### Identification of Living Cells

In this experiment, living cells were identified via calcein AM staining as previously described by Kajta et al. [26]. First, the cells grown on glass cover slips were washed with 10 mM PBS and then incubated with 2 mM calcein AM/PBS solution at room temperature for 10 min. As described above, qualitative analysis was conducted using a Leica DM IL LED Inverted Microscope (Leica Microsystems, Wetzlar, Germany) coupled with a CoolSnap camera (Vision Systems GmbH, Puchheim, Germany) with MetaMorph software (MetaMorph® Microscopy Automation & Image Analysis Software, Molecular Devices LLC, California, United States). Cells presenting bright green cytoplasm were considered living cells. In this case, the intensity of fluorescence was measured from entire photos using ImageJ software. The  final results are presented as a percentage of the control ± SEM.

### Identification of Apoptotic Cells

Detection of apoptotic cells was conducted with Hoechst-33342 staining after the initial experiment as described [27]. Cortical primary cells cultured on glass cover slips were washed with 10 mM phosphate-buffered saline (PBS) and incubated with Hoechst-33342 at a concentration of 0.6 mg/ml at room temperature for 5 min. Qualitative analysis was conducted using a Leica DM IL LED Inverted Microscope (Leica Microsystems, Wetzlar Germany) coupled with a CoolSnap camera (Vision Systems GmbH, Puchheim, Germany) with MetaMorph software (MetaMorph® Microscopy Automation & Image Analysis Software, Molecular Devices LLC, California, United States). Bright blue stained nuclei with condensed chromatin are widely recognized as a symptom of apoptosis. Fluorescence intensity was measured based on singular nuclei using ImageJ software. The  final results are presented as a percentage of the control ± SEM.

### mRNA Analysis Using qPCR

Total RNA from primary cell cultures was obtained using reagents from an RNeasy Mini Kit (Qiagen, USA) in accordance with the manufacturer’s protocol, as previously described by Wnuk et al. [28, 29]. The RNA was eluted in 40 µl of RNAse-free water. Then, the amount of RNA was assessed using a NanoDrop spectrophotometer at 260 nm, and the 260/280 ratio was obtained (ND/1000UV/VIS; Thermo Fisher Scientific, Waltham, MA, USA). A A260/A280 ratio of ~ 2.0 is considered to be an honest indicator of pure RNA. Subsequently, after isolation, the RNA extract was reverse transcribed to avoid freeze‒thaw cycles. Reverse transcription was conducted with a High-Capacity cDNA Reverse Transcription Kit in accordance with the manufacturer’s protocol using a CFX96 Real-Time System (Bio-Rad, Hercules, CA, USA). The collected cDNA was stored at − 20 °C overnight and subsequently subjected to quantitative polymerase chain reaction (qPCR). Amplification of the cDNA was conducted using FastStart Universal Probe Master containing TaqMan Gene Expression Assays specific for *Bax*, *Bcl2*, *Gsk3b*, *Fas*, *Fasl*, *Rbfox*, *Ache*, *Apoe*, *Chat*, *Bace1*, *Bace2*, *Mapt*, *App*, *Rcan1*, *Ide*, *Ngrn*, *Hprt1*, *Gapdh*, and *Actb.* In amplification, the following substances were used: a mixture containing 10 µl of FastStart Universal Probe Master, 1 µl of cDNA as template, 1 µl of the TaqMan Gene Expression Assay mix, and 8 µl of RNAse-free water in a total volume of 20 µl. qPCR was conducted using a CFX96 Real-Time system (Bio-Rad, USA), and the procedure consisted of intervals of varying temperatures as follows: 2 min at 50 °C, 10 min at 95 °C, 40 cycles of 15 s at 95 °C and 1 min at 60 °C. The obtained data were analyzed with the delta delta Ct method. The reference gene was chosen with the use of the following algorithms: geNorm, NormFinder, BestKeeper, and delta Ct; the three algorithms recommended hypoxanthine–guanine phosphoribosyltransferase (*Hprt1*).

### Western Blot Analysis

Once the experiment was concluded, neocortical cells underwent lysis in RIPA buffer with the addition of protease inhibitor. After lysis, the cells were sonicated to obtain homogenous solution, which was subsequently centrifuged at 15,000 × *g* for 20 min at 4 °C. To assess the protein concentration, a Bradford assay was conducted using Bradford reagent and bovine serum albumin as standards. Samples were then reconstituted and denatured in Laemmli sample buffer with β-mercaptoethanol. Subsequently, the samples underwent electrophoresis in 15-well [4–15%] SDS polyacrylamide gels (Bio-Rad, USA), and then, proteins were electrotransferred to PVDF membranes with the Bio-Rad Mini Trans-Blot apparatus as previously described [30, 31]. To block the non-specific binding sites, the PVDF membranes were incubated for 2 h with a solution of dried milk (5%) and Tween-20 (0.2%) in 0.02 M Tris-buffered saline (TBS). Next, the membranes were incubated overnight with primary antibodies at 4 °C. Primary antibodies were diluted using solution of Tween-20 and TBS in proportions as follows: anti-GAPDH mouse monoclonal antibody (diluted 1:3500), anti-BCL-2 rabbit polyclonal antibody (diluted 1:100), anti-BAX mouse monoclonal antibody (diluted 1:100), anti-GSK3β rabbit polyclonal antibody (diluted 1:700), anti-FAS mouse monoclonal antibody (diluted 1:80), and anti-FASL mouse monoclonal antibody (diluted 1:80). Next, the membranes were washed with Tween-20/TBS solution and incubated for 1 h with secondary antibodies coupled with horseradish peroxidase diluted in Tween-20/TBS solution 1:100 and 1:3500. Detection of the chemiluminescent signal was conducted employing BM (Chemiluminescence Blotting Substrate) and visualization using a Luminescence Image Analyzer Fuji-Las 4000 (Fuji, Japan). The intensity of the obtained bands was quantified with the MultiGauge V3.0 program (ScienceLab).

### *Bax* and *Bcl2 *Gene-Specific Methylation

Specific methylation of *Bax* and *Bcl2* genes was measured as described previously [32, 33]. Genomic DNA was obtained with GenElute™ Mammalian Genomic DNA Miniprep Kits, and the quantity of obtained DNA was assessed spectrophotometrically at wavelengths of 260 nm and 260/280 nm with a NanoDrop ND-1000 UV‒Vis Spectrophotometer (Thermo Fisher Scientific, Waltham, MA, USA). Then, denaturation followed by bisulfite conversion of GC-rich DNA was conducted with an EpiTect Bisulfite Kit obtained from Qiagen (Hilden, Germany). Samples were then eluted in a 10 µl volume and underwent qPCR (MethyLight) with an EpiTect MethyLight PCR Kit. The methylation regions in the *Bax* and *Bcl2* genes were verified in CpG hot spots in the 5′ flanking sequence (2000 bp). Methyl Primer Express Software 1.0 was employed to design primers for methylated and unmethylated target sequences. Fully methylated and fully unmethylated TaqMan probes were designed for the *Bax* and *Bcl2* promoters, and the internal reference set for the *Hprt1* gene was designed to control the input of DNA. For the EpiTect MethyLight assays, the specific TaqMan probes contained FAM™ as the reporter dye. The degree of methylation of each sample was calculated by taking the threshold cycles determined as percentage of methylation [%]: *C*_meth_ = 100/[1 + 2(∆Ct_meth_ − ∆Ct_unmeth_)].

### Immunofluorescence Staining

To visualize the cellular localization of the studied proteins and to confirm the neuronal character of cultured cells, immunofluorescence labeling followed by confocal microscopy was employed as previously described [34]. The cortical cells were cultured on glass coverslips and subjected to immunofluorescence labeling. Cells were fixed with a 4% paraformaldehyde solution in PBS for 15 min at room temperature and further incubated with blocking buffer, i.e., 5% normal donkey serum and 0.3% Triton X-100 in 0.01 PBS for 1 h. Subsequently, the neurons were incubated with the following primary antibodies for 24 h at 4 °C: anti-Aβ rabbit (diluted 1:50), anti-MAP2 mouse (1:100), anti-BAX mouse (1:50), anti-BCL2 rabbit (1:50), anti-FAS mouse (1:50), anti-FASL mouse (1:50), and anti-GSK3β rabbit (1:50). Next, the neocortical cells were incubated with secondary antibodies: Alexa Fluor Plus 488-conjugated goat anti-mouse IgG (1:200 and 1:600) and Alexa Fluor Plus 647-conjugated goat anti-rabbit IgG (1:200). Finally, the microscopic preparations were washed with PBS, mounted, and cover-slipped. For viewing the preparations, a Leica TCS SP8 WLL confocal laser scanning microscope (DMi8-CS, Leica Microsystem, Wetzlar, Germany) was employed.

### Fluoro-Jade C Staining

Fluoro-Jade C staining was used to assess the level of degenerating neurons as previously described [33, 35]. To conduct the staining, a stock solution with a concentration of 0.01% was prepared by diluting the Fluoro-Jade C in distilled water. The stock solution was then further diluted into a 0.005% working solution in Neurobasal medium. Then, the culture medium was removed from the 96-well plates, and the working solution was added at 100 µl per well. The plates were incubated for 45 min at room temperature. Then, the level of fluorescence was measured, with excitation = 490 nm and emission = 525 nm, using an Infinite M200 PRO microplate reader (Tecan Mannedorf, Switzerland). The results were analyzed by i-control software and presented as the percentage of the control ± SEM. Fluoro-Jade C is a green fluorescence dye. Microscopic images were obtained with a Leica DM IL LED Inverted Microscope (Leica Microsystems, Wetzlar, Germany). In this study, we used ImageJ to change the color to magenta.

### Neurite Outgrowth Staining

To assess neurite outgrowth, the Neurite Outgrowth Staining Kit was employed. First, the cells were washed with PBS, and then, the appropriate amount of 1X working Fix/Stain Solution was added to each well. Then, the cells were incubated for 15 min at room temperature. Next, the staining was visualized using a Leica TCS SP8 WLL confocal laser scanning microscope (DMi8-CS, Leica Microsystem, Wetzlar, Germany). The obtained images were analyzed with ImageJ, and the fluorescence intensity from entire images was measured. The data were normalized to the signal intensity of vehicle-treated cells and expressed as a percentage of the control ± SEM.

### ROS Activity Measurement

The ROS-Glo™ H_2_O_2_ Assay was used to assess the level of reactive oxygen species (ROS) in neocortical cells exposed to Aβ and PaPE-1. The assay was performed according to the manufacturer’s protocol. The bioluminescence was measured using a GloMax® Navigator Microplate Luminometer (Promega, Madison, WI, USA). The detected signal was proportional to the amount of H_2_O_2_ in cultured cells. The data were normalized to the signal intensity of vehicle-treated cells and expressed as a percentage of the control ± SEM.

### Assessment of Mitochondrial Membrane Potential

The JC-10 dye is a commonly employed fluorescent marker that forms aggregates (emitting red fluorescence) within mitochondria when the mitochondrial membrane potential is high. When mitochondrial membrane potential is decreased, JC-10 stays in the cytoplasm in monomeric form (green fluorescence). The assay was performed following the manufacturer’s protocol and fluorescence measurements were taken at Ex/Em = 490/525 nm and Ex/Em = 540/590 nm using an Infinite M200 PRO microplate reader (Tecan Mannedorf, Switzerland). The fluorescence intensity data were used to calculate the ratio, and the results were expressed as a percentage relative to the control, with the standard error of the mean (SEM). The data were normalized to the fluorescence intensity of control cells.

### Data Analysis

Statistical analysis was performed on raw data. The obtained results are presented as the mean absorbance, fluorescence or luminescence units per well containing 50,000 cells for the caspase activity assessment, JC-10, ROS activity and Fluoro-Jade C, mean fluorescence of the whole picture or single nuclei for calcein AM, Hoechst 33342 and neurite outgrowth staining, the fluorescence units per 1.5 million cells for qPCR and specific gene methylation, and the mean optical density per 10 µg of protein for western blot assays. To determine overall significance, an analysis of variance (ANOVA) was used. The differences between the control and experimental groups were defined with a post hoc Newman‒Keuls test that was preceded by Levene’s test for homogeneity.

Differences of statistical significance were indicated as follows: *p < 0.05, **p < 0.01, ***p < 0.001 (versus control cultures) and ^^^*p* < 0.05, ^^^^*p* < 0.01, ^^^^^*p* < 0.001 (versus cultures exposed to Aβ). The results are expressed as the mean ± SEM.

## Results

### Aβ is Present in Primary Neocortical Cell Cultures 30 h Post Application

**Fig. 1:**
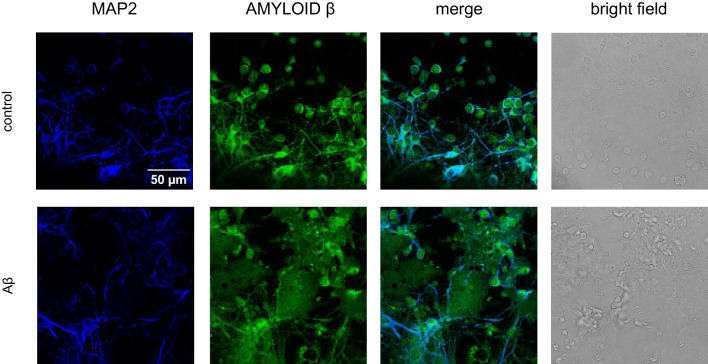
Immunofluorescent labeling of Aβ (green staining) and the neuronal marker MAP2 (blue staining) in primary neocortical cell cultures treated with 10 μM Aβ for 30 h. Bright-field images are also shown. Staining showed the intra- and extracellular distribution of Aβ in neuronal cell cultures

In the present study, primary neocortical cell cultures were exposed to Aβ (10 μM) for 30 h. Utilized concentration was chosen based on our previous study [22]. Immunofluorescence staining with the neuronal marker MAP2 (blue labeling) and Aβ (green labeling) showed the presence of preaggregated extracellular Aβ in Aβ-treated cultures. However, in control and Aβ-treated cells, endogenous intracellular Aβ was also present (Fig. 1).

### Aβ-Induced Cell Death Involves Apoptosis-Specific Pathways

**Fig. 2:**
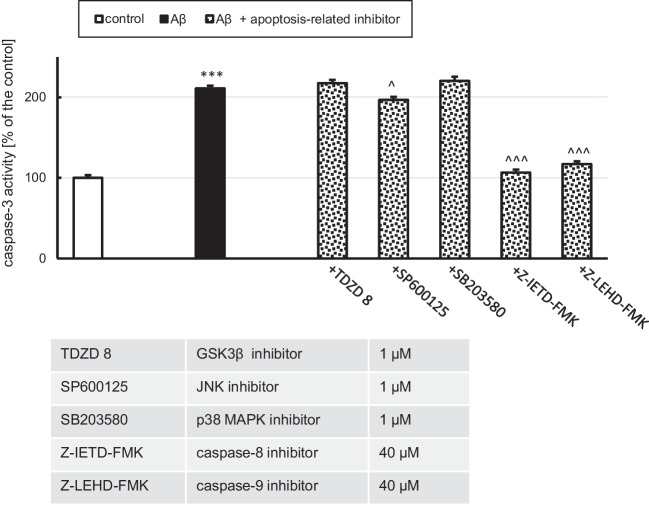
Aβ at a concentration of 10 μM increased caspase-3 activity. Inhibition of caspase-8 and caspase-9 prevented Aβ-induced caspase-3 overactivity, while JNK inhibition slightly attenuated the Aβ-induced effect. Each bar represents the mean ± SEM of three independent experiments, consisting of 10 replicates per group. ***p < 0.001 versus the control and ^*p* < 0.05, ^^^*p* < 0.001 versus Aβ-treated cells

In the present study, primary neocortical cell cultures exposed to Aβ (10 μM) for 30 h showed increased activity of caspase-3 (211% of the control), a hallmark of apoptosis (Fig. 2). Treatment with caspase-8 and caspase-9 inhibitors prevented caspase-3 activity elevation (106 and 117% of the control). Exposure to Aβ together with JNK inhibitor lowered caspase-3 elevation by 15%, while GSK3β and p38 MAPK inhibition had no effect on Aβ-induced caspase-3 activity (Fig. 2).

### PaPE-1 Inhibited Aβ-Induced Caspase-3 and Caspase-9 but Not Caspase-8 Overactivation

**Fig. 3:**
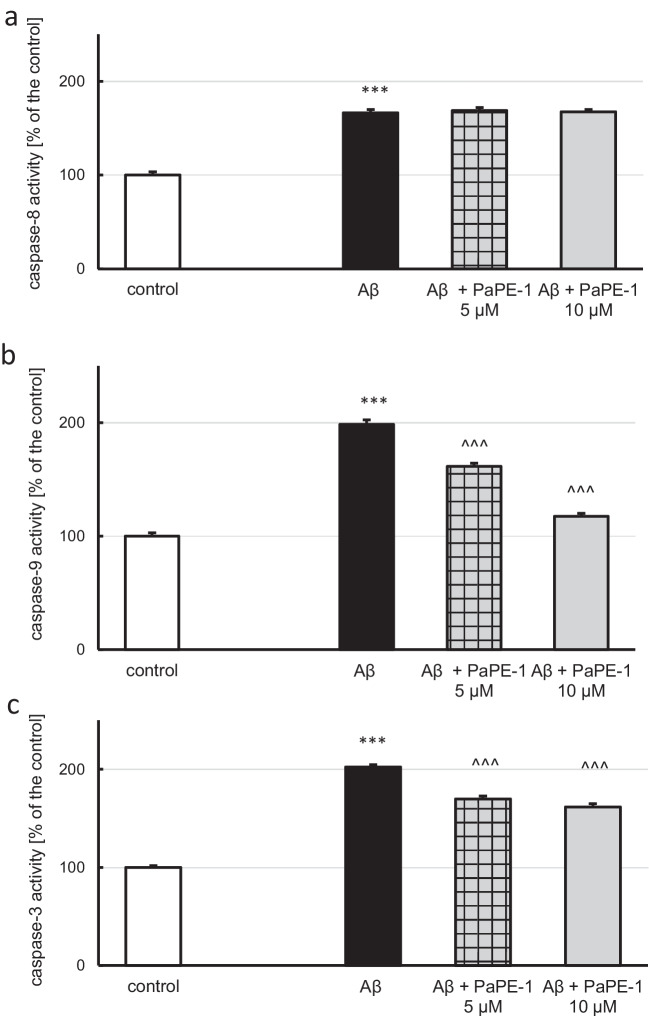
PaPE-1 (5 and 10 μM) partially reversed Aβ-induced caspase-9 (**b**) and caspase-3 activity (**c**) but did not impact caspase-8 elevation (**a**). Each bar represents the mean ± SEM of three independent experiments, consisting of 10 replicates per group. ***p < 0.001 versus the control and ^^^*p* < 0.001 versus Aβ-treated cells

In the current study, cells were exposed to Aβ (10 μM) for 24 h, and then, PaPE-1 was added for the following 6 h. Thirty hours of exposure to Aβ increased caspase-8 (Fig. 3a), caspase-9 (Fig. 3b), and caspase-3 (Fig. 3c) activities to 166, 198, and 202% of the control, respectively. Posttreatment with PaPE-1 (5 and 10 μM) decreased both caspase-3 (170 and 162% of the control) and caspase-9 activities (162 and 117% of the control) but had no impact on caspase-8 activity (Fig. 3). These results indicate that PaPE-1-induced neuroprotective effects include inhibition of caspase-9 and caspase-3. PaPE-1 did not alter the activity of any of the abovementioned enzymes under control conditions (Supplementary material Table S1).

### PaPE‑1 Restored Cell Viability Decreased by Exposure to Aβ and Reversed Aβ-Induced Apoptotic Chromatin Condensation, but It Did Not Affect ROS Activity and Mitochondrial Membrane Potential

**Fig. 4:**
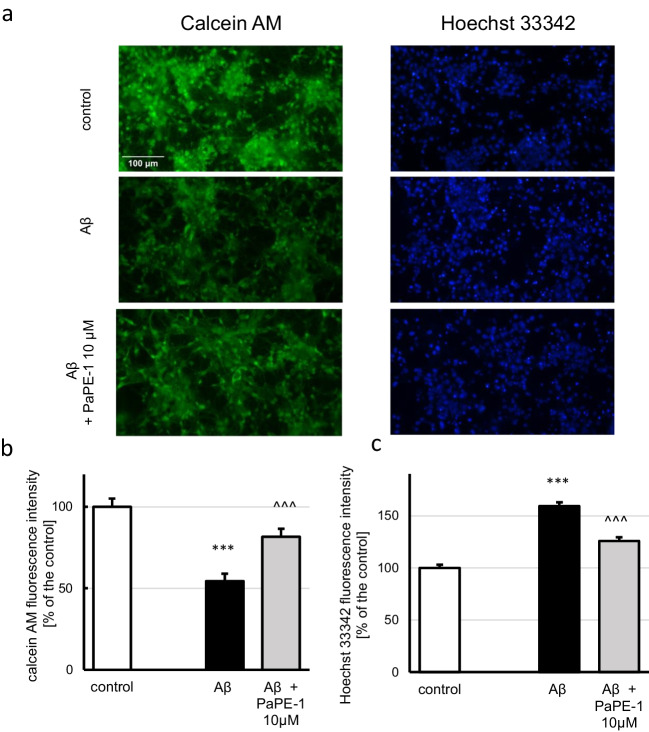
Fluorescent labeling of viable cells using calcein AM (green stain) and cell nuclei using Hoechst 33342 (blue stain) (**a**). PaPE-1 (10 μM) partially reversed the Aβ-induced decrease in cell viability (**b**) and increase in chromatin condensation (**c**). Each bar represents the mean ± SEM of the mean fluorescence intensity measured from 15 whole photos per group in calcein AM staining or 30 nuclei per picture with five pictures per group in Hoechst 33342 staining. ***p < 0.001 versus the control and ^^^*p* < 0.001 versus Aβ-treated cells

Staining with calcein AM showed that treatment with 10 μM Aβ decreased cell viability to 55% of the control. After treatment with PaPE-1 (10 μM), cell viability reached approximately 80% of the control, i.e., PaPE-1 increased this parameter by 25% (Fig. 4a, b). Hoechst 33342 enables visualization of condensed chromatin—another characteristic of apoptosis. Exposure to Aβ resulted in an increased number of bright-blue stained nuclei (159% of the control) that was partially reversed by posttreatment with PaPE-1 (126% of the control) (Fig. 4a, c). The staining provided additional evidence of extensive apoptosis in response to Aβ. In the control conditions, PaPE-1 altered neither cell viability nor apoptotic chromatin condensation (Supplementary material Table S1).

In addition, we did not observe any change in reactive oxygen species (ROS) activity in response to either Aβ (10 μM) or PaPE-1 (10 μM; Supplementary material Fig. S1). In contrast, mitochondrial membrane potential decreased in Aβ-treated cells, but application of PaPE-1 (5 and 10 μM) did not evoke changes of this parameter (Supplementary material Fig. S2).

### PaPE-1 Altered Aβ-Induced Changes in the Expression of Apoptosis-Specific Factors

**Fig. 5:**
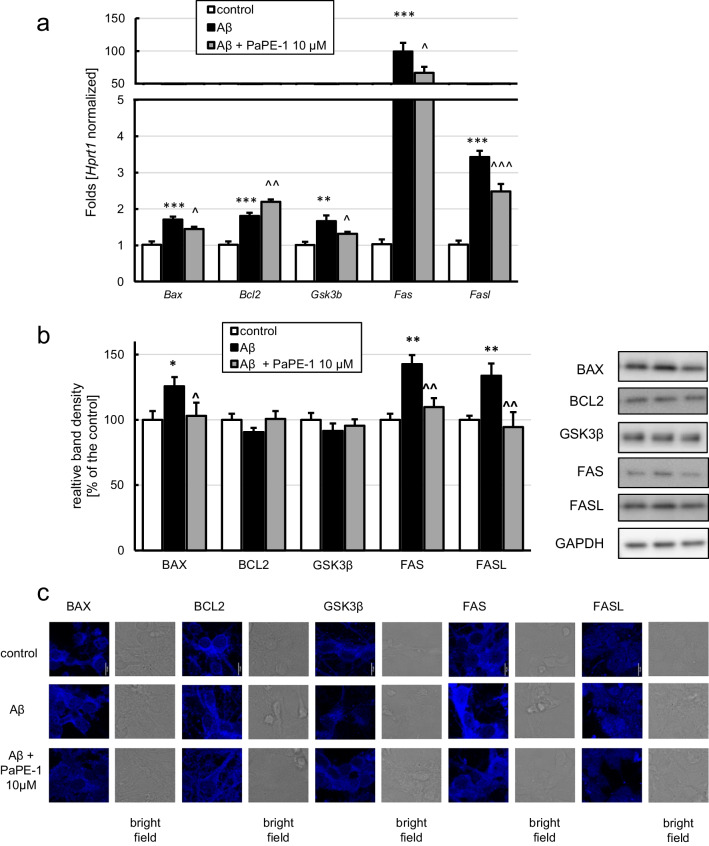
PaPE-1 (10 μM) affected Aβ-increased mRNA expression (**a**) and protein levels (**b**) of apoptosis-specific factors. Each bar represents the mean ± SEM of three independent experiments, consisting of five replicates per group. **p < 0.01 and ***p < 0.001 versus the control cultures; ^*p* <0.05, ^^*p* < 0.01 and ^^^*p* < 0.001 versus Aβ-treated cells. Immunofluorescent labeling of BAX, BCL2, GSK3β, FAS, and FASL (**c**) confirmed the results obtained using western blotting. Scale bar equals 10 μm in all images

Exposure to Aβ (10 μM) resulted in an increase in the expression of all investigated apoptosis-specific genes, i.e., *Bax* (1.71-fold), *Bcl2* (1.81-fold), *Gsk3b* (1.66-fold), *Fas* (99.24-fold), and *Fasl* (3.43-fold; Fig. 5a). PaPE-1 at a concentration of 10 μM partially reversed Aβ-induced changes in the expression of all the abovementioned proapoptotic genes, i.e., *Bax* (from 1.71-fold to 1.45-fold), *Gsk3b* (from 1.66-fold to 1.32-fold), *Fas* (from 99.24-fold to 66.50-fold), and *Fasl* (from 3.43-fold to 2.48-fold; Fig. 5a)*.* PaPE-1 also influenced anti-apoptotic *Bcl2*, causing a further increase in its expression from 1.81-fold to 2.20-fold (Fig. 5a).

Concerning the protein levels, Aβ elevated the expression of BAX (126% of the control), FAS (143% of the control), and FASL (134% of the control) (Fig. 5b). Posttreatment with PaPE-1 normalized Aβ-induced changes in BAX, FAS, and FASL levels (Fig. 5b). Levels of BCL2 and GSK3β were not altered in response to either Aβ or PaPE-1 (Fig. 5b). Immunofluorescent labeling of BAX, BCL2, GSK3β, FAS, and FASL confirmed the results obtained using western blotting (Fig. 5c).

In the control conditions, PaPE-1 increased the mRNA expression of *Fasl* 1.66-fold (Supplementary material Table S2) and did not alter any other apoptosis-specific mRNA levels. At the protein level, PaPE-1 decreased BAX expression (70% of the control) and increased GSK3β expression to approximately 120% of the control (Supplementary material Table S3).

### Aβ and PaPE-1 Altered the Methylation Rate of the *Bax* and *Bcl2* Genes

**Fig. 6:**
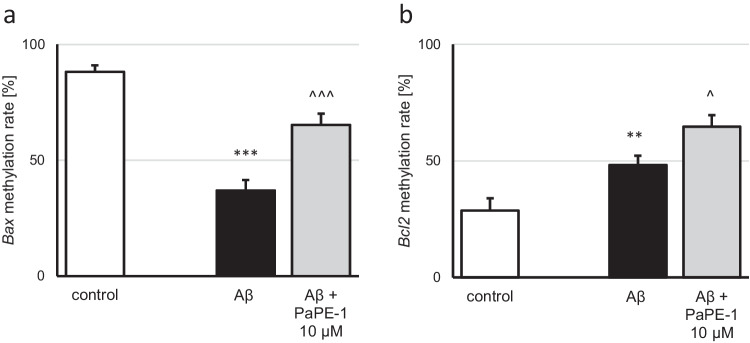
The methylation rates of the *Bax* (**a**) and *Bcl2* (**b**) genes were altered by both Aβ (10 μM) and posttreatment with PaPE-1 (10 μM). The results are presented as the mean ± SEM. There were three independent experiments, consisting of five replicates per group. **p < 0.01 and ***p < 0.001 compared to the control group; ^*p* < 0.05 and ^^^*p* < 0.001 compared to the cultures exposed to Aβ

The basic methylation rate of the *Bax* gene was 88%, and PaPE-1 (10 μM) did not affect the rate (Fig. 6a, Supplementary material Table S4). Aβ (10 μM) decreased the *Bax* methylation rate (37%), while posttreatment with PaPE-1 partially reversed the observed change (65%) (Fig. 6a).

The *Bcl2* methylation rate in the control condition was 29%. This value was elevated to 48% after application of Aβ. PaPE-1 increased both the control and Aβ-affected *Bcl2* methylation rates to 69% and 65%, respectively (Fig. 6b, Supplementary material Table S4).

### PaPE‑1 Partially Reversed Aβ‑Evoked Neurodegeneration

**Fig. 7:**
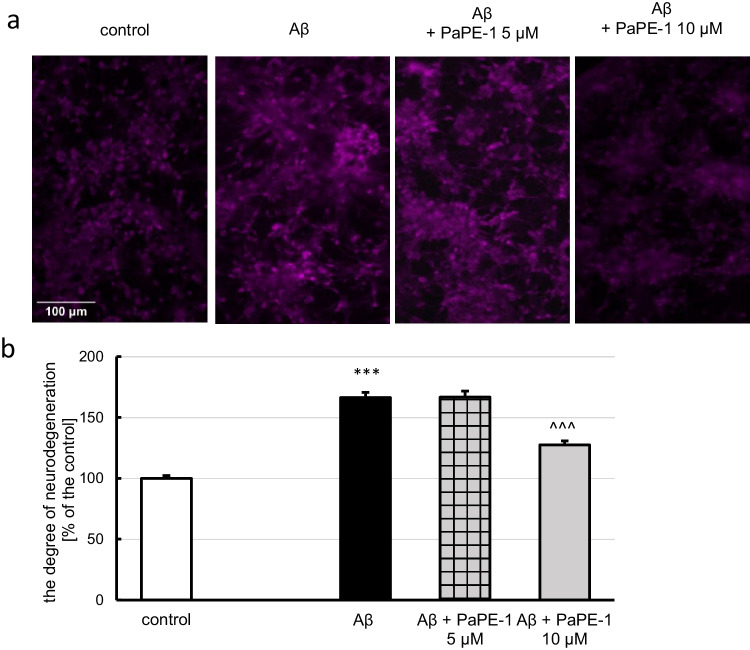
Fluoro-Jade C was used to stain degenerating neurons (**a**, **b**). PaPE-1 decreased the Aβ-induced increase in the degree of neurodegeneration. Each bar represents the mean ± SEM of three independent experiments, consisting of 10 replicates per group. ***p < 0.001 versus the control and ^^^*p* < 0.001 versus Aβ-treated cells

Fluoro-Jade C staining showed that Aβ (10 μM) elevated the degree of neurodegeneration to 166% of the control. Posttreatment with PaPE-1 at a concentration of 10 μM effectively decreased the parameter to 130% of the control. The 5 μM PaPE-1 appeared ineffective (Fig. 7). PaPE-1 did not alter the parameter in the control conditions (Supplementary material Table S1).

### PaPE-1 Restored Neurite Outgrowth, Which Decreased in Response to Aβ

**Fig. 8:**
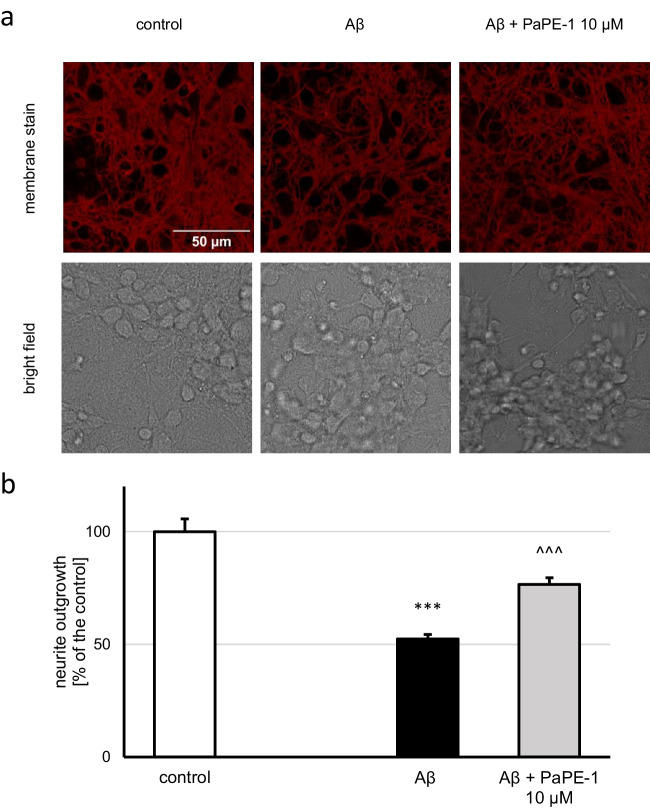
PaPE-1 (10 μM) partially reversed the Aβ-induced decrease in neurite outgrowth (**a**, **b**). Each bar represents the mean ± SEM of the mean fluorescence intensity measured from 23 to 25 photos per group. ***p < 0.001 versus the control and ^^^*p* < 0.001 versus Aβ-treated cells

Aβ (10 μM) application decreased neurite outgrowth to 52% of the control, and posttreatment with PaPE-1 (10 μM) increased the parameter to 77% of the control (Fig. 8a, b). Neurite outgrowth was not altered by treatment with PaPE-1 under control conditions (Supplementary material Table S1).

### PaPE-1 Reversed Aβ-Induced Changes in AD-Related Gene Expression

**Fig. 9:**
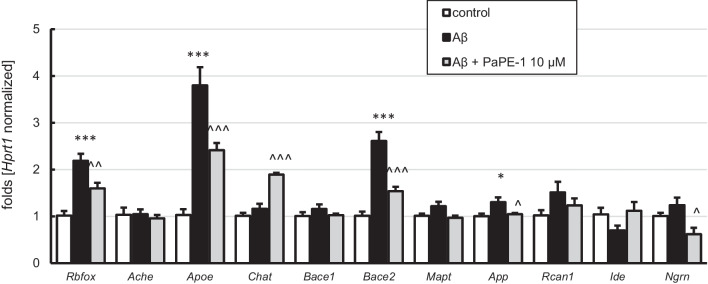
Aβ (10 μM) and PaPE-1 (10 μM) affected the mRNA expression levels of AD-related genes. Each bar represents the mean ± SEM of three independent experiments, consisting of five replicates per group. *p < 0.05 and ***p < 0.001 versus the control cultures; ^*p* < 0.05, ^^*p* < 0.01, and ^^^*p* < 0.001 versus Aβ-treated cells

Treatment with Aβ (10 μM) increased the expression of *Rbfox* (2.2-fold), *Apoe* (3.8-fold), *Bace2* (2.6-fold), and *App* (1.3-fold) (Fig. 9). Cells posttreated with PaPE-1 (10 μM) showed partial reversal of all of the abovementioned changes. PaPE-1 induced *Rbfox* decrease from 2.2-fold to 1.6-fold, *Apoe* from 3.8-fold to 2.2-fold, *Bace2* from 2.6-fold to 1.5 fold, and *App* from 1.3-fold to 1.0 fold (Fig. 9). In addition, in Aβ-treated cells PaPE-1 elevated the expression of *Chat* (1.9-fold) and reduced the expression of *Ngrn* (0.6-fold). The levels of *Ache*, *Bace1*, *Mapt*, *Rcan1*, and *Ide* did not change in response to either Aβ or PaPE-1 (Fig. 9). Interestingly, in the control conditions, PaPE-1 reduced the expression of *Bace1* and did not alter other AD-related mRNAs (0.03-fold) (Supplementary material Table S2).

## Discussion

Targeting the non-nuclear estrogen receptor (ER) signaling, mainly via mERα and mERβ, has been postulated as novel therapeutic strategy for central nervous system pathologies. Recently, we showed that PaPE-1, which selectively activates ER non-nuclear signaling pathways, elicited neuroprotection in a cellular model of AD [22]; however, in the study, the ligand was applied at the same time as Aβ. In the present study, to increase the translational value of the research, we assessed the neuroprotective capacity and mechanisms of action of PaPE-1 in a posttreatment paradigm, i.e., 24 h after initiating Aβ-induced neurotoxicity. In this study, primary neocortical cell cultures treated with preaggregated Aβ_1-42_ (10 µM) showed the presence of extracellular Aβ_1-42_, confirming the adequacy of the AD model used. Extracellular storage of Aβ is a common feature of AD pathology and causes synaptic loss and neurodegeneration [36]. The Aβ protein is a primary risk factor for AD known to accumulate and aggregate in the brains of AD patients. Cerebral organoids from Dutch-type cerebral amyloid angiopathy (D-CAA) patients exhibited Aβ accumulation, thus confirming the potential of cerebral organoids as an in vitro disease model [37]. Similar to our study, aggregated Aβ was observed in primary cultures of rat hippocampal pyramidal neurons that were treated with preaggregated Aβ [38]. Aβ aggregates were also seeded in mouse primary neurons treated with APP/PSEN1 brain lysates, as detected with an antibody against Aβ_42_ and immunofluorescence labeling [39].

We are the first to show in this study that a 24-h delayed posttreatment with PaPE-1 decreased the degree of Aβ-induced neurodegeneration, restored neurite outgrowth, and inhibited the expression of AD-related genes, i.e., *Rbfox*, *Apoe*, *Bace2*, *App*, and *Ngrn*, except for *Chat*, which was stimulated. There are no relevant data to compare our results with, since except for our study no attempt has been made to selectively activate non-nuclear ER signaling pathways, particularly mERα and mERβ, and to measure AD-related parameters. Furthermore, in most studies, the neuroprotective capacities of new ER ligands have been tested as pre- or cotreatment paradigms but not as posttreatment paradigm, as in the present study. The only relevant study was based upon 1-h posttreatment with a single dose of 4-estren-3α,17β-diol (estren), a non-classical ER pathway activator, which inhibited the loss of cholinergic cortical projections and attenuated Aβ_1-42_-induced learning deficits in ovariectomized mice [40]. This is why we discussed the neuroprotective effect of posttreatment with PaPE-1 against Aβ_1-42_-induced neurotoxicity in the context of other ER ligands without dissecting non-nuclear ER signaling pathways specific effects.

Similar to our results, a phytoestrogen genistein that displays strong binding affinity for ERα and has the selective estrogen receptor modulator (SERM) property appeared to inhibit Aβ-induced neurotoxicity in hippocampal neurons and to improve brain function [41, 42]. In addition, selective modulators of ERβ were effective in protecting against AD pathology in transgenic models of the disease [43], and treatment with hydroxytyrosol acetate caused ERβ-dependent cognitive improvement in APP/PS1 transgenic mice [44]. A phytoSERM that contains genistein, daidzein, and *S*-equol and preferentially targets ERβ was found to preserve cognitive function in women with genetic risk modulators for AD, i.e., mitochondrial haplogroup and *APOE* genotype [45]. A functional link between ER signaling and AD could rely on APOE-mediated modulation of *ESR1* that involves CEBPB/ATF4, miR-155-5p, or miR-1-3p [46]. Therefore, the cited studies confirm the rationale for undertaking our research; however, they do not indicate the exclusive participation of non-nuclear ER signaling pathways in neuroprotection against AD as our present study does.

We demonstrated that the AD-attributed effects were accompanied by activation of apoptosis-dependent caspases (caspase-3, caspase-8, and caspase-9) and JNK kinases, as evidenced using relevant inhibitors. Previously, we also showed the involvement of apoptosis in the response of neuronal cells to Aβ, including activation of caspase-3, loss of mitochondrial membrane potential, and induction of apoptosis-specific factors [22]. Many other studies have also supported the process of apoptosis as an initial trigger of AD both in vivo and in vitro [4, 47]. In this study, posttreatment with PaPE-1 elicited anti-apoptotic effects by inhibiting Aβ-induced caspase-3 and caspase-9 activities as well as attenuating apoptotic nuclei formation, thus preventing neuronal cell death. The anti-apoptotic properties of ER agonists have been supported by a wide variety of studies, including the beneficial actions of *S*-equol (ERβ agonist) on platelet mitochondria cytochrome oxidase (COX) activity in AD women [48] and neuroprotective effects exerted by a newly synthesized benzopyran FMDB (R-9-(4fluorophenyl)-3-methyl-10,10-hydrogen-6-hydrogen-benzopyran) on cognition, neurogenesis, and apoptosis in APP/PS1 transgenic mice with ERβ knockdown [49]. Furthermore, ERα is known to promote nonamyloidogenic APP processing via the MAPK/ERK pathway [50], and ginsenoside was shown to regulate ERα phosphorylation to protect against AD pathology [51]. Our results indicate that PaPE-1-mediated anti-apoptotic effects involve caspase-9 inhibition. Tamayev et al. [52] showed that inhibition of caspase-9 activity rescues both synaptic plasticity and memory deficits in Danish dementia knock-in mice, implicating caspase-9 in the pathogenesis of the disease and suggesting that PaPE-1 by reducing caspase-9 activity could be a valid therapeutic approach to treating human dementias.

In addition, posttreatment with PaPE-1 downregulated the Aβ-affected mRNA expression of apoptosis-specific factors such as *Bax*, *Gsk3b*, *Fas*, and *Fasl*, except for *Bcl2*, which was upregulated. In parallel, PaPE-1 decreased the protein levels of BAX, FAS, and FASL, which were elevated due to Aβ treatment, as detected by western blotting and immunofluorescence labeling. Interestingly, PaPE-1 also decreased the control level of BAX, but increased the control level of GSK3β. Since GSK3β activity is determined by its phosphorylation on specific sites, and only the active GSK3β is a measure of apoptosis, we do not interpret PaPE-1-evoked increase in the control level of GSK3β as a proapoptotic effect. GSK3β inhibition was observed in response to trehalose, which upregulated ERα and ERβ and protected APP/PS1 mice against dietary advanced glycation end product (dAGE)-induced neurotoxicity and cognitive impairment [53]. Our results are also in line with the ERα-mediated anti-apoptotic effects of adenosine against Aβ_25-35_-induced brain damage [54] and of formononetin in AD patients [55], as well as the ER/PI3K/Akt-mediated effect of naringenin against Aβ_25-35_-caused damage in neuronally differentiated PC12 cells [56].

In our model of AD, Aβ caused *Bax* hypomethylation and *Bcl2* hypermethylation, which suggests opposite regulation of the expression levels of the genes and related proteins. Accordingly, the PaPE-1-evoked decrease in the BAX/BCL2 ratio corresponds to increased methylation of the *Bax* gene and implies a decreased BAX protein level. However, *Bcl2* gene hypermethylation and intact BCL2 protein levels suggest other PaPE-1-dependent mechanisms to control Aβ-induced apoptosis. BCL2 upregulation has been unexpectedly detected in response to hypoxia, ischemia, and neurodegenerative diseases and was explained as a compensatory mechanism preventing neurons from acute or chronic injury [21, 22, 33, 35, 57–59]. BCL2 dysregulation may be related to the dual role of autophagy during neurodegeneration, depending on its interaction with the process of apoptosis and abilities to bind and inhibit Ca^2+^ flux of inositol-1,4,5-trisphosphate receptors (IP3Rs) and ryanodine receptors (RyRs). Paradoxically, the anti-apoptotic function of BCL2 may be neutralized by sensitizers such as BAD, BIK, NOXA, PUMA, HRK, and BMF, which in turn may predispose cells to apoptosis. In AD patients, BCL2 immunoreactivity in neurons increases in parallel with increasing disease severity [60]. However, in AD patients with confirmed neurofibrillary degeneration, BCL2 immunoreactivity decreases, which supports the unclear role of this protein in preventing Aβ-induced apoptosis, including PaPE-1-evoked *Bcl2* gene hypermethylation observed in the present study.

Summing up, current basic study proposes a novel therapeutic approach for AD that relies on a posttreatment with the newly designed PaPE-1 which selectively activates ER non-nuclear signaling pathways, inhibits the expression of AD-related genes and apoptosis process that involves enhanced DNA methylation of specific genes, and in these ways protects from Aβ-induced neurodegeneration.

### Supplementary Information

Below is the link to the electronic supplementary material.

Supplementary file1 (DOCX 33.1 KB)

## Data Availability

The data that support the findings of this study are available from the corresponding author upon reasonable request. Some data may not be made available because of privacy or ethical restrictions.
