# Supplementary material for: Posttreatment with PaPE-1 Protects from Aβ-Induced Neurodegeneration Through Inhibiting the Expression of Alzheimer’s Disease-Related Genes and Apoptosis Process That Involves Enhanced DNA Methylation of Specific Genes
Source: Mol Neurobiol. 2023 Dec 8;61(7):4130–45. doi: 10.1007/s12035-023-03819-5 (PMC11236864; doi:10.1007/s12035-023-03819-5)
Supplement: Supplementary file 1 — Supplementary file1 (DOCX 33.1 KB) [file 12035_2023_3819_MOESM1_ESM.docx]

Posttreatment with PaPE-1 protects from Aβ-induced neurodegeneration through inhibiting the expression of Alzheimer’s disease-related genes and apoptosis process that involves enhanced DNA methylation of specific genes

*Molecular Neurobiology*

Bernadeta A. Pietrzak-Wawrzyńska, Agnieszka Wnuk, Karolina Przepiórska-Drońska, Andrzej Łach, Małgorzata Kajta*

Laboratory of Neuropharmacology and Epigenetics, Department of Pharmacology, Maj Institute of Pharmacology, Polish Academy of Sciences, Smętna Street 12, Krakow, 31-343, Poland.

* corresponding author

kajta@if-pan.krakow.pl

Table S1

|  | control | control + 5 μM PaPE-1 | control + 10 μM PaPE-1 |
| --- | --- | --- | --- |
| **caspase-8 activity**  [% of the control] | 100.00 ± 3.47 | 95.12 ± 3.13 | 91.34 ± 3.31 |
| **caspase-9 activity**  [% of the control] | 100.00 ± 2.81 | 95.77 ± 4.31 | 94.58 ± 4.75 |
| **caspase-3 activity**  [% of the control] | 100.00 ± 1.59 | 93.27 ± 1.78 | 94.89 ± 2.86 |
| **calcein AM**  [% of the control] | 100.00 ± 7.06 | - | 91.73 ± 5.13 |
| **Hoechst 33342**  [% of the control] | 100.00 ± 5.82 | - | 93.53 ± 5.86 |
| **Fluoro-Jade C**  [% of the control] | 100.00 ± 0.76 | 100.27 ± 0.71 | 99.05 ± 0.77 |
| **neurite outgrowth membrane stain** [% of the control] | 100.00 ± 5.67 | - | 91.74 ± 7.57 |

**Tab S1**

The effects of PaPE-1 (5 and 10 μM) on caspase-8, -9, and -3 activity, the degree of neurodegeneration (Fluoro-Jade C staining), cell viability (calcein AM), apoptotic chromatin condensation (Hoechst 33342), and neurite outgrowth (membrane stain). The results are presented as a percentage of the control. Each value represents a mean ± SEM number of 10 to 20 replicates.

Table S2

| Folds [Hprt1 normalized] | control | control + 10 μM PaPE-1 |
| --- | --- | --- |
| **Bax** | 1.02 ± 0.09 | 1.07 ± 0.02 |
| **Bcl2** | 1.01 ± 0.09 | 1.05 ± 0.06 |
| **Gsk3b** | 1.01 ± 0.08 | 1.17 ± 0.12 |
| **Fas** | 1.03 ± 0.13 | 1.89 ± 0.14 |
| **Fasl** | 1.02 ± 0.11 | 1.66 ± 0.06 ** |
| **Rbfox** | 1.02 ± 0.10 | 1.26 ± 0.16 |
| **Ache** | 1.03 ± 0.15 | 1.29 ± 0.12 |
| **Apoe** | 1.03 ± 0.12 | 1.10 ± 0.08 |
| **Chat** | 1.01 ± 0.06 | 0.82 ± 0.03 |
| **Bace1** | 1.01 ± 0.08 | 0.03 ± 0.00 *** |
| **Bace2** | 1.01 ± 0.09 | 0.67 ± 0.08 |
| **Mapt** | 1.01 ± 0.04 | 0.96 ± 0.05 |
| **App** | 1.01 ± 0.05 | 0.81 ± 0.11 |
| **Rcan1** | 1.02 ± 0.11 | 1.93 ± 0.39 |
| **Ide** | 1.04 ± 0.14 | 1.08 ± 0.19 |
| **Ngrn** | 1.01 ± 0.07 | 0.90 ± 0.19 |

**Tab S2**

The effects of PaPE-1 (10 μM) on mRNA expression. The results are presented as a mean ± SEM. There were 3 independent experiments, consisting of 5 replicates per group. ** p < 0.01, and *** p < 0.001 compared to the control group.

Table S3

| Protein level [GAPDH normalized] | control | control + 10 μM PaPE-1 |
| --- | --- | --- |
| BAX | 100.00 ± 6.60 | 70.25 ± 5.62 ** |
| BCL2 | 100.00 ± 4.68 | 100.04 ± 6.00 |
| GSK3β | 100.00 ± 5.29 | 118.70 ± 6.69 * |
| FAS | 100.00 ± 4.69 | 68.72 ± 7.90 |
| FASL | 100.00 ± 3.17 | 81.17 ± 3.20 |

**Tab S3**

The effects of PaPE-1 (10 μM) on protein levels. The results are presented as a mean ± SEM. There were 3 independent experiments consisting of 5 replicates per group. ** p < 0.01, and *** p < 0.001 compared to the control group.

Table S4

| Methylation rate | control | control + 10 μM PaPE-1 |
| --- | --- | --- |
| Bax | 88.14 ± 5.35 | 80.42 ± 6.74 |
| Bcl2 | 28.67 ± 5.35 | 69.18 ± 3.98 *** |

**Tab S4**

The methylation rate of *Bax* and *Bcl2* genes in response to PaPE-1(10 μM) in the control conditions. The results are presented as a mean ± SEM. There were 3 independent experiments consisting of 5 replicates per group. *** p < 0.001 compared to the control group.

**Fig. S1** In utilized model no changes in ROS activity have been observed in response to Aβ (10 μM) or PaPE-1 (10 μM). Each bar represents the mean ± SEM of 3 independent experiments, consisting of 10 replicates per group.

**Fig. S2** Aβ at a concentration of 10 μM decreased mitochondrial membrane potential. PaPE-1 (5 and 10 μM) did not impact mitochondrial membrane potential in control and Aβ-treated cells. Each bar represents the mean ± SEM of 3 independent experiments, consisting of 10 replicates per group. ***p < 0.001 versus the control
